# Progressive changes in synapses and glial cells in *App^NL-G-F^* mice, a model of Alzheimer’s disease

**DOI:** 10.1093/braincomms/fcaf484

**Published:** 2025-12-12

**Authors:** Megan Tomlin, Marina Podpolny, Patricia C Salinas

**Affiliations:** Department of Cell and Developmental Biology, University College London, London WC1E 6BT, UK; Department of Cell and Developmental Biology, University College London, London WC1E 6BT, UK; Department of Cell and Developmental Biology, University College London, London WC1E 6BT, UK

**Keywords:** synapse degeneration, astrocytes, microglia, *hAPP^NL-G-F/NL-G-F^* Alzheimer’s disease

## Abstract

It is well documented that synapse loss correlates with cognitive decline in Alzheimer’s disease. However, the mechanisms that contribute to synapse loss remain poorly understood. Studies have shown that amyloid-β directly signals to neurons to trigger changes in synaptic function leading to the subsequent loss of synapses. Other studies have demonstrated that glial cells directly target synapses in Alzheimer’s disease. In this study, we determine the temporal relationship between changes in synapses and glial cells (microglia and astrocytes) in the NL-G-F knock-in mouse model of Alzheimer’s disease. We evaluated synapse number and histological changes in glial cells in the hippocampus of NL-G-F mice using confocal microscopy across three timepoints, 2, 5, and 9 months, compared to their wild-type littermates. Using real-time quantitative PCR, we also evaluated molecular changes in glial cells. At 2 months of age, when very few amyloid-β plaques are present, inhibitory synapse number was transiently increased by more than 50% in NL-G-F mice, accompanied by a small increase in the microglial marker, *Cx3cr1,* and considerable changes in astrocyte markers, including a decreased level of *Thbs1/2*. At 5 months, when amyloid-β plaque load is notable, excitatory synapse number was decreased immediately proximal to plaques, whereas inhibitory synapse number was no different between NL-G-F and wild-type mice. At the cellular level, changes in microglia and astrocytes were also observed in NL-G-F mice in regions closely surrounding plaques. From 5 months, PCR analyses indicated marked and progressive changes in microglia and astrocyte markers, including increased *Trem2* and *Gfap* expression. By 9 months, changes in excitatory synapse number and microglia at the cellular level were exacerbated, with evident synapse loss extending up to 30 µm away from plaques. Together, our data show that inhibitory synapses are the earliest change in NL-G-F mice occurring concomitantly with molecular changes in glial cells and preceding substantial plaque deposition, excitatory synapse loss, and glial cellular alterations.

## Teaser

Tomlin *et al*. examined the temporal changes in synapses and glial cells in a mouse model of Alzheimer’s disease. They found that inhibitory synapses are the earliest change in NL-G-F mice with concomitant molecular changes in glial cells, preceding substantial plaque deposition, excitatory synapse loss, and glial cellular alterations.

## Graphical Abstract

**Graphical Abstract:**
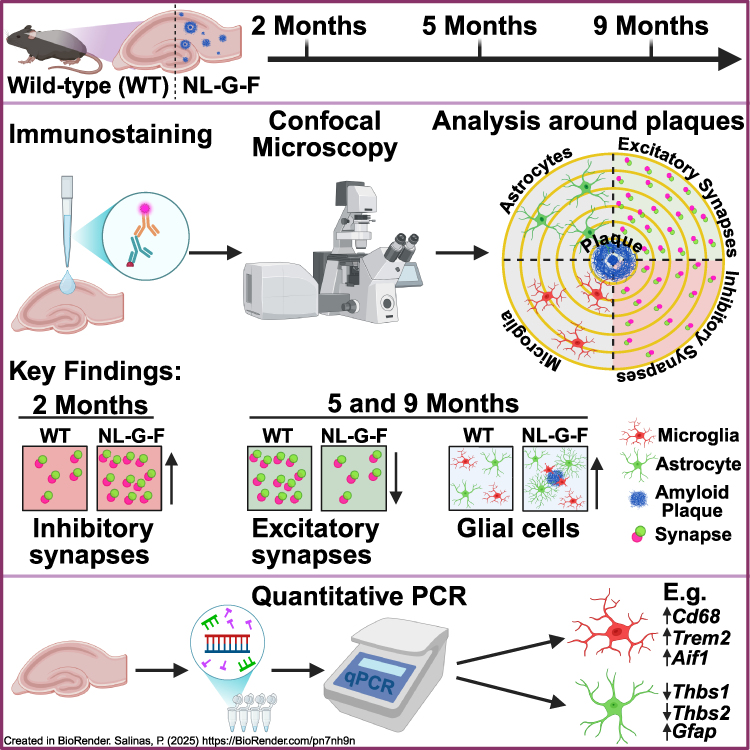

## Introduction

Alzheimer’s disease is a progressive neurodegenerative condition, characterized by cognitive decline and an impaired ability to form and retain new memories. Memory impairment in Alzheimer’s disease has been correlated with the loss of synapses in the human brain.^1-4^ Several studies suggest that synapse loss can occur before the appearance of insoluble amyloid beta (Aβ) plaques^4,5^ and that soluble Aβ oligomers are the toxic amyloid species that affect synaptic function and integrity.^5-7^ However, studies in human and mouse Alzheimer’s disease models have shown clear synapse loss in close proximity to plaques and has therefore been suggested that plaques act as a reservoir for toxic Aβ oligomers.^8-10^ *In vitro* and *in vivo* studies have revealed that synapses become dysfunctional and disassemble before significant evidence of cell death in Alzheimer’s disease.^11-13^ Thus, synapses are susceptible to the toxic exposure to Aβ oligomers, present around plaques, and are considered one of the earlier cellular targets in Alzheimer’s disease.

Several studies including high-throughput analyses and genome-wide association studies (GWAS), suggest a critical role of glial cells, inflammation, and changes in vasculature function in Alzheimer’s disease pathogenesis.^6,14-17^ Astrocytes and microglia have a significant impact on the function and stability of synapses.^15,18-20^ Several studies have demonstrated profound changes in glial cells during the progression of Alzheimer’s disease, particularly around Aβ plaques.^14^ *In vivo* experiments have demonstrated that microglial cells contribute to synapse degeneration induced by Aβ.^21,22^ However, the role of astrocytes is less clear as these cells have both deleterious and protective effects on synapses in both health and disease.^6,23,24^ It remains unclear whether synapse loss correlates in time with changes in glial cells in Alzheimer’s disease. Establishing the temporal relationship of these events could focus efforts to identify the primary targets that contribute to synapse vulnerability.

Many Alzheimer’s disease mouse models have been generated, which have greatly contributed to our current understanding of the molecular, cellular, and functional changes that occur during Alzheimer’s disease progression.^25^ Some of these models are based on the overexpression of mutant Amyloid Precursor Protein (APP) associated with familial Alzheimer’s disease, providing invaluable insight into Alzheimer’s disease pathogenesis (https://www.alzforum.org/). However, legitimate concerns were raised around the impact of the ectopic and/or high expression levels of these mutant APP variants in mouse Alzheimer’s disease overexpression models, leading to the generation of knock-in (KI) mouse models carrying mutant APP variants at the endogenous APP locus.^26,27^ Interestingly, many of the KI models exhibit similar phenotypes as those observed in overexpression models, whilst exhibiting physiological levels of APP expression.^25,26^ Thus, KI models are emerging as the ideal tool to study Alzheimer’s disease pathogenesis.

Here we investigated whether synapse degeneration correlates with changes in glial cells in the *hAPP^NL-G-F/NL-G-F^* (NL-G-F) knock-in Alzheimer’s disease mouse model, which carries the humanized form of APP with the Swedish, Iberian and Arctic mutations, leading to Aβ overproduction.^27^ We compared NL-G-F homozygous mice to their wild-type (WT) littermates at different ages (2, 5, and 9 months). We used confocal microscopy to determine histological changes in synapse number and glial cells (microglia and astrocytes) in the *stratum radiatum* (SR) of the CA1 region of the hippocampus, as synapse loss in this area has been well documented in human Alzheimer’s disease.^2^ At 2 months, when no or very few Aβ plaques are present in NL-G-F mice,^27-29^ we found a transient increase in inhibitory synapse number with no changes in excitatory synapse number or microglia and astrocyte cell number, coverage, or intensity. From 5 months, when a substantial Aβ plaque load was present in the hippocampus,^27-29^ no changes in inhibitory synapse number were detected. However, a significant loss of excitatory synapses was observed proximal to plaques in NL-G-F mice, and this phenotype was further enhanced at 9 months. From 5 months onwards, we also observed significant changes in microglia and astrocyte cell number, coverage, and intensity of IBA1 (microglia) or GFAP (astrocyte) inside and proximal to plaques. To further identify changes in glial cells, we performed reverse transcription and quantitative RT-PCR (RT-qPCR) analyses from bulk hippocampal tissue of NL-G-F mice compared to their WT littermates. This approach revealed changes in the expression of microglia and astrocyte markers from 2 months old, before changes in excitatory synapses were evident. In conclusion, NL-G-F mice displayed early changes in hippocampal inhibitory synapse number, accompanied with molecular changes in both microglia and astrocytes, and before substantial plaque deposition. In contrast, changes in excitatory synapses followed plaque deposition and were concomitant with histological and molecular changes in glial cells that become more pronounced with age in this Alzheimer’s disease model.

## Materials and methods

### Mice

All animal procedures were conducted in accordance with the Animals Scientific Procedures Act UK (1986), the ethical standards at University College London (UCL) and ARRIVE guidelines. Animals were kept in ventilated cages under a 12-h light-dark cycle and *ad libitum* access to food and water.

NL-G-F mice were genotyped using ear biopsies and the following primers: 5′-ATCTCGGAAGTG AAGATG-3′, 5′-ATCTCGGAAGTGAATCTA-3′, 5′-TGTAGATGAGAACTTAAC-3′ and 5′-CGTATAATGTATGCTATACGAAG-3′.^27^ Mice were maintained on a C57BL/6J background.

Both male and female WT and NL-G-F mice were used across different ages (2, 5, and 9 months). The genotype, age, number and sex of mice used within each experimental data set is indicated as follows: 2-month excitatory synapse immunostaining *n* = 9 WT mice (4 females; 5 males) and *n* = 8 NL-G-F mice (3 females; 5 males); 2-month inhibitory synapse immunostaining *n* = 9 WT mice (4 females; 5 males) and *n* = 9 NL-G-F mice (3 females; 6 males); 2-month glial cell immunostaining *n* = 3 WT mice (1 female; 2 males) and *n* = 3 NL-G-F mice (3 males); 2-month qPCRs *n* = 6 WT mice (4 females; 2 males) and *n* = 6 NL-G-F mice (3 females; 3 males); 5-month excitatory synapse, inhibitory synapse and glial cell immunostaining and qPCRs *n* = 6 WT mice (1 female; 5 males) and *n* = 6 NL-G-F mice (1 female; 5 males); 9-month excitatory synapse, inhibitory synapse and glial cell immunostaining and qPCRs *n* = 6 WT mice (4 females; 2 males) and *n* = 6 NL-G-F mice (1 female; 5 males).

### Tissue processing for immunofluorescence microscopy

Brain tissue was collected from WT and NL-G-F mice and fixed overnight in 4% PFA before being immersed in 30% sucrose solution for cryopreservation and frozen using dry-ice chilled isopentane. Brain tissues were then embedded in OCT and cryopreserved. The tissue was sectioned in 30 µm thick sagittal hippocampal sections using a Leica cryostat (Model CM1850).

Immunofluorescence staining of free-floating brain slices was performed as previously described.^30,31^ Briefly, brain slices were washed three times with PBS and then incubated in blocking/permeabilization buffer containing 10% donkey serum and 0.5% Triton X-100 in PBS for 3–6 h at room temperature. Brain slices were then incubated with the relevant primary antibodies (Table 1) diluted in blocking/permeabilization buffer overnight at 4°C, followed by three washes with PBS and then incubated for a further 2–3 h with the corresponding secondary antibodies diluted in blocking/permeabilization buffer at room temperature. Slices were then DAPI stained (1:10 000, Invitrogen) and washed a further two times in PBS before mounting on glass slides with Fluoromount-G mounting medium (Southern Biotech). For staining of dense-core plaques, Thioflavin-S (Thio-S) (Sigma T1892-25G) was added to slices after secondary antibody staining for other markers as indicated above. Slices were washed three times with PBS before washing with 70%, then 80% ethanol for 1 min and incubated in Thio-S diluted in 80% ethanol for 15 min. Following this, slices were washed in 80%, then 70% ethanol for 1 min before a final three washes with distilled water and then mounted as described above.

**Table 1:** Table of antibodies

Antibody	Species	Dilution	Company (catalogue #)
Anti-bassoon	Mouse	1:500	Novus Biologicals, Cat# NB120-13249
Anti-gephyrin	Rabbit	1:500	Synaptic Systems, Cat# 147 008
Anti-GFAP	Chicken	1:500	Millipore, Cat# AB5541
Anti-homer1	Chicken	1:1000	Synaptic Systems, Cat# 160 006
Anti-IBA1	Guinea Pig	1:500	Synaptic Systems, Cat# 234 308
Anti-LAMP1 (1D4B) (Supernatant)	Rat	1:200	Developmental Studies Hybridoma Bank, RRID: AB_528127
Anti-vGAT	Guinea Pig	1:500	Synaptic Systems, Cat# 131 004
Anti-6E10	Rabbit	1:1000	Novus Biologicals, Cat# NBP2-62566

### Image acquisition and analyses

All images were acquired on a Leica SP8 confocal microscope using a 63× (1.40 Numerical Aperture) oil objective, unless otherwise stated. For synapse and glial cell image acquisition, three images were acquired per brain slice from two to three slices per animal in the CA1 SR of the hippocampus. For all WT mice and 2-month-old NL-G-F mice, regions of interest (ROIs) were selected by measuring 100 µm below the cell body layer and imaging three consecutive ROIs. For 5- and 9-month-old NL-G-F mice, ROIs were selected around plaques. For synaptic puncta, each image stack consisted of 9 equidistant planes, 0.25 µm apart. For microglia and astrocytes, each image stack consisted of 22 equidistant planes, 1 µm apart.

**Figure 1:**
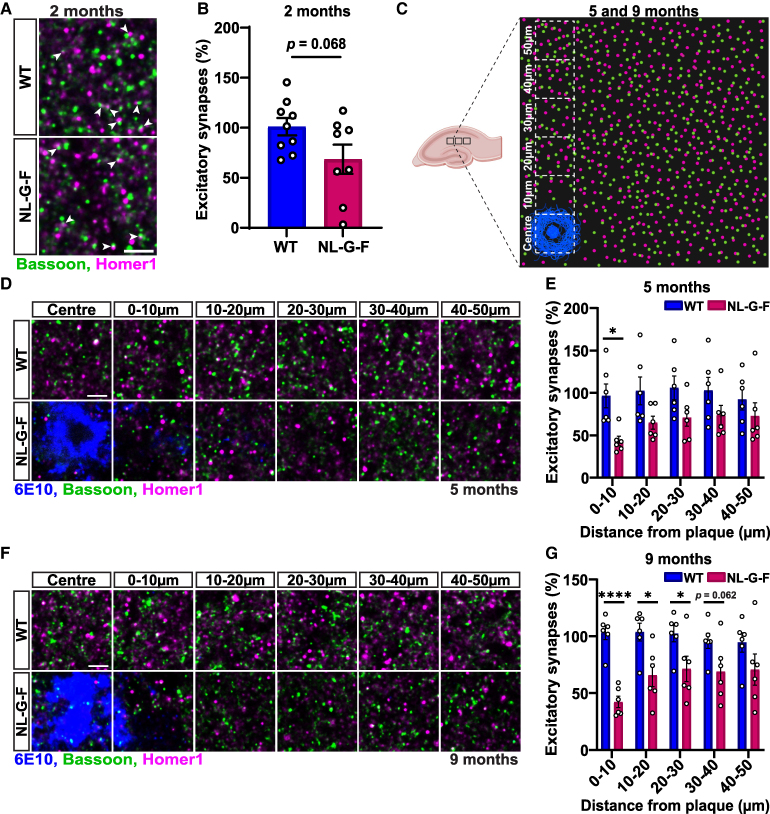
**Progressive loss of excitatory synapses following Aβ plaque formation in the hippocampus of NL-G-F mice.** (**A)** Confocal representative images of excitatory synapses (bassoon in green and homer1 in magenta) from the CA1 SR of 2-month-old WT and NL-G-F mice. Arrows point to synapses. Scale bar: 2 µm. (**B)** Quantification of excitatory synapse number shown as a percentage relative to WT in 2-month-old mice. Synapses were defined by the co-localization of the pre- and post-synaptic markers. Two-tailed unpaired *t*-test. *n* = 9 WT mice, *n* = 8 NL-G-F mice. **(C)** Schematic diagram depicting how image acquisition and synapse quantification around 6E10 Aβ plaques (blue) was performed in the CA1 SR region of the hippocampus in brain slices. Created in BioRender. Salinas, P. (2025) https://BioRender.com/oaoz47y. **(D)** Confocal representative images of excitatory synapses in 10 µm increments up to 50 µm away from 6E10^+^ Aβ plaques (blue) in the CA1 SR of 5-month-old WT and NL-G-F mice. Scale bar: 2 µm. **(E)** Quantification of excitatory synapse number shown as a percentage relative to WT in 5-month-old mice. Non-parametric analysis of longitudinal data in factorial experiments followed by a post-hoc Dunn’s test. Significant genotype-by-distance interaction: *F*(4, 40) = 4.015, *P* = 0.023. WT versus NL-G-F: 0–10 µm *P* = 0.026. *n* = 6 per genotype. **(F)** Confocal representative images of excitatory synapses in 10 µm increments up to 50 µm away from 6E10^+^ Aβ plaques in the CA1 SR of 9-month-old WT and NL-G-F mice. Scale bar: 2 µm. **(G)** Quantification of excitatory synapse number shown as a percentage relative to WT in 9-month-old mice. Repeated measures two-way ANOVA followed by a post-hoc Tukey test. Significant genotype-by-distance interaction: *F*(4, 40) = 12.99, *P* < 0.0001. WT versus NL-G-F: 0–10 µm *P* < 0.0001, 10–20 µm *P* = 0.014, 20–30 µm *P* = 0.045. *n* = 6 per genotype. Graphs represent synapse number per 200 µm^3^. Images are presented in an XY plane. 2–3 brain slices were quantified per animal. *P < 0.05, **** *P* < 0.0001. Data are represented as mean ± SEM. Each data point represents one animal. Homer1, homer scaffold protein 1; WT, wild-type.

Synapse quantification was performed using Volocity 3D Image Analysis software version 6.5.1 (Quorum Technologies). To analyse synapse number, six 10 µm^2^ × 10 µm2 consecutive crops were taken from the plaque centre up to 50 µm away (Fig. 1C) or a random area within the ROI for WTs. Custom thresholding protocols were then applied to crops from the plaque border (0–10 µm), excluding the core/centre, to detect synapses. Synapses were defined by the co-localization of pre- and post-synaptic puncta. Synapse number was normalized to the WT mean value across all distances.

Quantification of astrocytes and microglia was performed using ImageJ FIJI (NIH). A customized macro was used to first threshold an image and detect the plaque of interest. For all WT mice and 2-month-old NL-G-F mice, a centrally placed circle was added to each image to serve as a reference plaque. Subsequently, an ROI was drawn around the plaque, and radiating concentric circles in 10 µm increments up to 50 µm away from the plaque. Each image was individually thresholded to detect plaques. A threshold was then manually applied to images to measure intensity and percent coverage of IBA1 for microglia or GFAP for astrocytes within plaques and in each concentric circle radiating from plaques. The same threshold was used across all images to measure mean IBA1 or GFAP intensity and percent coverage. Number of microglia and astrocytes was counted manually in concentric circles emanating from Aβ plaques and calculated by dividing by the corresponding concentric circle area. Cells were counted based on the cell body, DAPI co-localized with IBA1 for microglia and DAPI co-localized with GFAP for astrocytes. For microglia, cells were counted in 10 µm increments up to 50 µm away from plaques. For astrocytes, due to their larger nature, concentric circles of 0–25 µm and 25–50 µm were drawn around plaques instead. Where a cell body was split evenly between the border of two concentric circles, the cell was counted within the inner most circle to avoid double counting. Microglia and astrocyte datasets were normalized to the WT mean values across all distances.

### RNA extraction, RT-qPCR analyses

RNA was extracted from WT and NL-G-F mouse hippocampal tissue using TRIzol (Thermo Fisher Scientific) and the DirectZol RNA MiniPrep Kit (Zymo Research), as previously described.^32^ Briefly, hippocampal tissue was first homogenized in TRIzol. Chloroform was then added to the homogenized tissue, vortexed and left at room temperature for 10 min. Samples were then centrifuged at 4°C and the upper transparent aqueous phase containing RNA was extracted and transferred into new Eppendorf tubes. To extract RNA, the DirectZol RNA MiniPrep Kit (Zymo Research, cat# R2052) was used following the manufacturer’s instructions. RNA concentration and purity was measured using a Nanodrop ND-100 (Thermo Fisher Scientific). Following this, retrotranscription to first-strand cDNA was performed using the RevertAid H Minus First Strand cDNA Synthesis kit (Thermo Fisher Scientific, cat# K1632) as per the manufacturer’s instructions. 10 ng of RNA was used to perform qPCR’s using GoTaq qPCR Master Mix (Promega, cat# A6002) in a CFX96 Bio-Rad instrument with the following protocol: 95°C for 3 min followed by 40 cycles of denaturing at 96°C and 30 s at 55°C before annealing/extension at 60°C. Samples were run in triplicate. Primers (Table 2) were purchased from Sigma-Aldrich and used at a final concentration of 0.5 μM. Fold change in messenger RNA (mRNA) expression was calculated using the comparative threshold cycle (Ct) method. Average Ct values were obtained using the CFX Manager software version 3.1 (Bio-Rad). Gene expression was normalized to the expression of housekeeping genes (*Gapdh, GusB* and *Pgk1*).

**Table 2:** Primer sequences

Gene	Forward primer sequence	Reverse primer sequence
*Aif1*	GGATCAACAAGCAATTCCTCGA	CTGAGAAAGTCAGAGTAGCTGA
*Cd68*	GGGCTCTTGGGAACTACAC	GTACCGTCACAACCTCCCTG
*Chi3l1*	GCTTTGCCAACATCAGCAGCGA	AGGAGGGTCTTCAGGTTGGTGT
*Cx3cr1*	GAGTATGACGATTCTGCTGAGG	CAGACCGAACGTGAAGACGAG
*C1qa*	CCGGGTCTCAAAGGAGAGAG	AGATTCCCCTGGGTCTCCT
*C1qbp*	GTGAAGAGGAGCCCTCACA	CCGATCTCGTCCTCAGGATA
*C3*	CCAGCTCCCCATTAGCTCTG	GCACTTGCCTCTTTAGGAAGTC
*Gapdh*	CGTCCCGTAGACAAAATGGT	TCAATGAAGGGGTCGTTGAT
*Gfap*	AGAAAACCGCATCACCATTC	CCTTCTGACACGGATTTGGT
*Gusb*	GGTTTCGAGCAGCAATGGTA	GCTGCTTCTTGGGTGATGTC
*Itgam*	TGGCCTATACAAGCTTGGCTTT	AAAGGCCGTTACTGAGGTGG
*Il-1β*	TGAAATGCCACCTTTTGACA	GGGTCCGTCAACTTCAAAGA
*Il-6*	GTTCTCTGGGAAATCGTGGA	TTCTGCAAGTGCATCATCGT
*Lcn2*	CCAGTTCGCCATGGTATTTT	CACACTCACCACCCATTCAG
*Pgk1*	TACCTGCTGGCTGGATGG	CACAGCCTCGGCATATTTCT
*P2ry12*	TACCCTACAGAAACACTCAAG	GCTGAATCTGAAGGATATGAG
*Slc1a2*	TTCCAAGCCTGGATCACTGCTC	GGACGAATCTGGTCACACGCTT
*Sparcl1*	CCTTCAGATGAGGGCAACTC	CAGTGAGGTTTCCCTTGTGG
*Thbs1*	AACTGTGACCCTGGACTTGC	CGCTGAAGTCCACAGCATTA
*Thbs2*	ACCTGAGGAATGCCCTGTG	TGCCACCTGTAGGCAGTGTA
*Tlr4*	TTCTTCTCCTGCCTGACACC	TGTCATCAGGGACTTTGCTG
*Tmem119*	GTGTCTAACAGGCCCCAGAA	AGCCACGTGGTATCAAGGAG
*Tnf-α*	ATGGCCTCCCTCTCATCAGT	GGCTACAGGCTTGTCACTCG
*Trem2*	CTGGAACCGTCACCATCACTC	CGAAACTCGATGACTCCTCGG

### Statistical analyses

Sample size was based on previous published work from our lab.^30,31^ In all graphs, *n*-numbers correspond to the number of animals unless otherwise specified. No inclusion/exclusion criteria were set; no animals were excluded. GraphPad Prism version 10.1.0 and RStudio version 2024.04.2 were used for statistical analyses. ROUT test was used to determine statistical outliers and dataset normality was tested using the Shapiro-Wilk test in GraphPad Prism. All outliers were included in the datasets. Statistical analyses were conducted using a 95% confidence level. For parametric data, when comparing between two groups, an unpaired two-tailed Student’s T-test was used and for comparisons between more than two groups, a repeated measures two-way ANOVA followed by a post-hoc Tukey’s multiple comparisons test was used in GraphPad Prism. For non-parametric data, when comparing between two groups, an unpaired two-tailed Mann-Whitney U test was used in GraphPad Prism and for comparisons between more than two groups, a non-parametric analysis of longitudinal data in factorial experiments was performed in RStudio, using the R package nparLD. The *F1-LD-F1* design was followed to test group and time effects, and interaction. Data presented in graphs are displayed as mean ± SEM. In all figures, *P*-values are depicted as: *P < 0.05, **P < 0.01, ***P < 0.001, ****P < 0.0001.

## Results

### Progressive changes in excitatory synapses in the hippocampus of NL-G-F mice

Previous studies have shown that NL-G-F mice exhibit changes in synaptic markers.^27,31,33^ However, changes in synapse number during disease progression have not been fully characterized in relation to changes in glial cells at the cellular and molecular level. Here, we examined changes in excitatory synapse number in the SR of the hippocampus, where axons from CA3 pyramidal neurons make synapses with CA1 pyramidal dendrites. We evaluated NL-G-F mice compared to control WT littermates at different ages, taking careful consideration of the distance of synapses in relation to Aβ plaques. We identified excitatory synapses based on the co-localization of pre- and post-synaptic markers, Bassoon and Homer1 respectively, using confocal microscopy.

We first examined 2-month-old animals, when no or very few Aβ plaques are present in the hippocampus. We observed a trend towards a decrease in excitatory synapse number in NL-G-F mice compared to control WT mice, but this change was not statistically significant (Fig. 1A and B). Next, we examined 5-month-old mice when a considerable Aβ plaque load is observed. Previous studies in mouse Alzheimer’s disease models and in human Alzheimer’s disease brains have demonstrated that synapses are more affected proximal to plaques.^8,10,31^ We therefore examined the number of excitatory synapses at increasing distances from Aβ plaques (labelled with 6E10 antibody) in 10 µm increments, up to 50 µm away from the edge of Aβ plaques (Fig. 1C; Supplementary Fig. 1A). Quantification of confocal images at 5 months (Fig. 1D) showed a significant decrease in the number of excitatory synapses at 0–10 µm from plaques compared to WT controls (Fig. 1E). By 9 months old, the hippocampus exhibits substantial Aβ plaque load in NL-G-F mice.^27,28^ At this age, we observed a more robust decrease in excitatory synapse number in NL-G-F mice when compared to WT mice (Fig. 1F). Quantification showed a statistically significant decrease in the number of excitatory synapses at 0–30 µm from the plaque (Fig. 1G). Our analyses demonstrate a progressive loss of excitatory synapses, with a clear effect proximal to Aβ plaques, at different ages in NL-G-F mice following notable Aβ plaque deposition.

### Inhibitory synapses are transiently affected in NL-G-F mice

**Figure 2:**
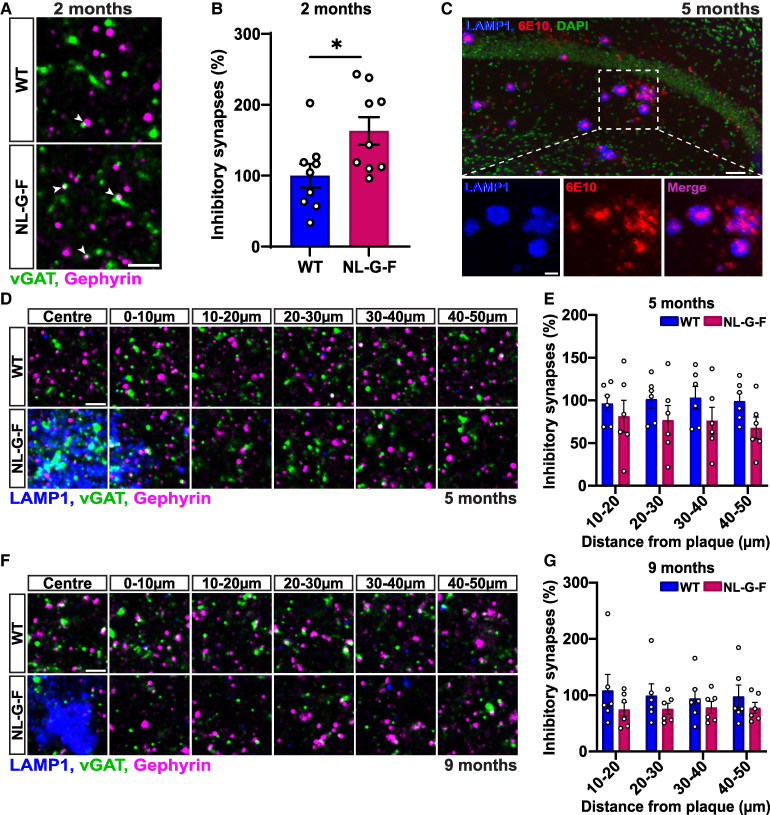
**Inhibitory synapse number increases prior to substantial Aβ plaque deposition in the hippocampus of NL-G-F mice. (A)** Confocal representative images of inhibitory synapses (vGAT in green and gephyrin in magenta) from the CA1 SR of 2-month-old WT and NL-G-F mice. Arrows point to synapses. Scale bar: 2 µm. **(B)** Quantification of inhibitory synapse number shown as a percentage relative to WT in 2-month-old mice. Synapses were defined by the co-localization of the pre- and post-synaptic markers. Two-tailed unpaired *t*-test: *P* = 0.025. *n* = 9 per genotype. **(C)** Confocal z-stack representative images of LAMP1 and 6E10 immunostaining in the hippocampus of a 5-month-old NL-G-F mouse. Top: Whole image, 20× objective no zoom, labelled with LAMP1 (blue), 6E10 (red), and DAPI (green) (scale bar 50 µm). Bottom: Zoomed in pictures from the 20× full image; LAMP1 (left), 6E10 (middle) and LAMP1/6E10 merge (right) (scale bar: 20 µm). (**D)** Confocal representative images of inhibitory synapses in 10 µm increments up to 50 µm away from LAMP1^+^ plaques (blue) in the CA1 SR of 5-month-old WT and NL-G-F mice. Scale bar: 2 µm. **(E)** Quantification of inhibitory synapse number from 10 µm up to 50 µm away from Aβ plaques shown as a percentage relative to WT in 5-month-old mice. Repeated measures two-way ANOVA followed by a post-hoc Tukey test. No significant interaction. *n* = 6 per genotype. **(F)** Confocal representative images of inhibitory synapses in 10 µm increments up to 50 µm away from LAMP1^+^ plaques (blue) in the CA1 SR of 9-month-old WT and NL-G-F mice. Scale bar: 2 µm. **(G)** Quantification of inhibitory synapse number from 10 µm up to 50 µm away from Aβ plaques shown as a percentage relative to WT in 9-month-old mice. Non-parametric analysis of longitudinal data in factorial experiments followed by a post-hoc Dunn’s test. No significant interaction. *n* = 6 per genotype. Graphs represent synapse number per 200µm.^3^ Images are presented in an XY plane unless otherwise stated. 2–3 brain slices were quantified per animal. *P < 0.05. Data are represented as mean ± SEM. Each data point represents one animal. DAPI, 4′,6-diamidino-2-phenylindole; LAMP1, lysosomal-associated membrane protein 1; vGAT, vesicular GABA transporter; WT, wild-type.

Whilst changes in excitatory synapses have been investigated in NL-G-F mice, the impact on inhibitory synapses in NL-G-F mice have not been well documented. We therefore examined inhibitory synapse number at different ages in NL-G-F mice, in relation to their distance to Aβ plaques. To evaluate inhibitory synapse number, we determined the co-localization of the pre- and post-synaptic markers, vGAT and Gephyrin respectively. Due to antibody compatibility issues with gephyrin and the Aβ plaque marker, 6E10, we used an antibody against LAMP1 (lysosomal-associated membrane protein 1) to label Aβ plaques (Fig. 2C).^34^ However, we found that the antibody against LAMP1 partially co-localized with large cellular aggregates labelled with vGAT, present within and at short distances from plaques. To overcome this issue, we measured changes in inhibitory synapse number from a 10 µm distance from plaques rather than from 0 to 10 µm.

At 2 months old, before substantial plaque deposition, we found a significant increase (>50%) in the number of inhibitory synapses in the CA1 SR of the hippocampus in NL-G-F mice when compared to control WT mice (Fig. 2A and B). As we have done previously to evaluate excitatory synapses, we measured inhibitory synapse number in 5- and 9-month-old mice in 10 µm increments, up to 50 µm away from Aβ plaques labelled with LAMP1 (Supplementary Fig. 1B). At these two ages, we observed no statistically significant changes in inhibitory synapse number between WT and NL-G-F mice (Fig. 2D–G). These results demonstrate that NL-G-F mice exhibit an early transient increase in inhibitory synapse number, but this effect is not sustained when Aβ plaque burden is increased.

### Changes in microglia during synapse degeneration

Changes in glial cells have been observed during the progression of Alzheimer’s disease, from the alteration of specific microglial and astrocytic markers, to changes in their morphology and function.^35^ Functional studies have shown that microglia play a critical role in the engulfment and subsequent loss of synapses in Alzheimer’s disease models.^15,22,36^ We therefore analysed microglial cells in the SR of the CA1 region of the hippocampus in NL-G-F mice compared to WT controls across different ages. To assess changes in microglia, we labelled cells with IBA1, measuring cell number (number of IBA1 and DAPI positive cells in each region of interest), coverage (area labelled with IBA1 within a given region of interest), and mean intensity of IBA1 signal.@@

**Figure 3:**
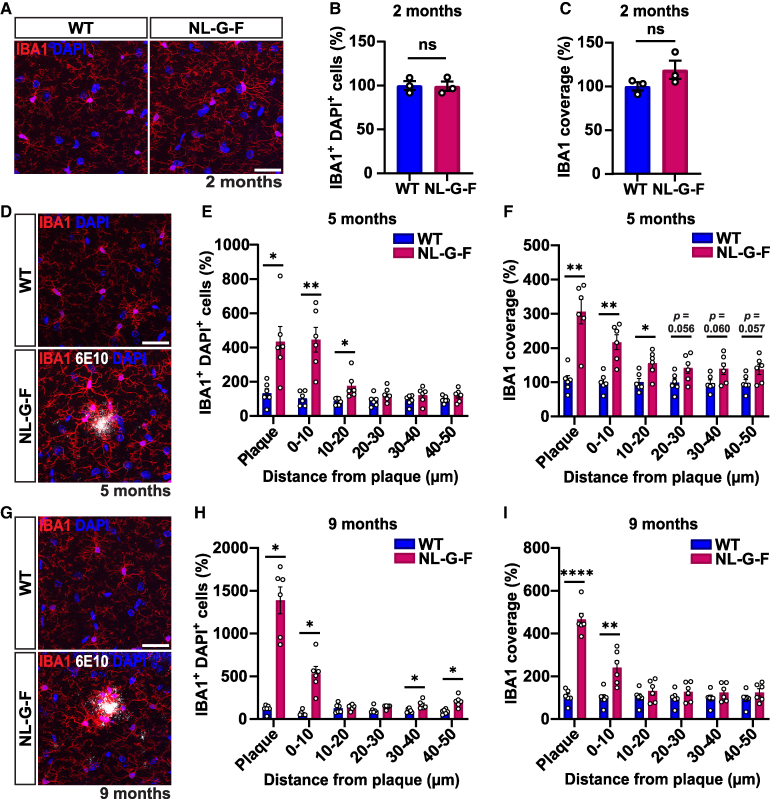
**Progressive changes in microglia number and coverage in the hippocampus of NL-G-F mice with age. (A)** Confocal representative images of microglial cells from the CA1 SR of 2-month-old WT and NL-G-F mice. Scale bar 30 µm. **(B-C)** Quantification of microglial cell number (**B**) and coverage (**C**) shown as a percentage relative to WT in 2-month-old mice. Two-tailed unpaired *t*-test. *n* = 3 per genotype. (**D)** Confocal representative images of microglia around 6E10^+^ Aβ plaques (white) from the CA1 SR of 5-month-old WT and NL-G-F mice. Scale bar 30 µm. **(E)** Quantification of microglia number at increasing distances from 6E10^+^ Aβ plaques shown as a percentage relative to WT in 5-month-old mice. Repeated measures two-way ANOVA followed by a post-hoc Tukey test. Significant genotype-by-distance interaction: *F*(5, 50) = 7.151, *P* < 0.0001. WT versus NL-G-F: plaque *P* = 0.017, 0–10 µm *P* = 0.004, 10–20 µm *P* = 0.029. *n* = 6 per genotype. (**F)** Quantification of microglial cell coverage at increasing distances from 6E10^+^ amyloid plaques shown as a percentage relative to WT in 5-month-old mice. Repeated measures two-way ANOVA followed by a post-hoc Tukey test. Significant genotype-by-distance interaction: *F*(5, 50) = 32.10, *P* < 0.0001. WT versus NL-G-F: plaque *P* = 0.002, 0–10 µm *P* = 0.002, 10–20 µm *P* = 0.014. *n* = 6 per genotype. **(G)** Confocal representative images of microglial cells around 6E10^+^ Aβ plaques from the CA1 SR of 9-month-old WT and NL-G-F mice. Scale bar 30 µm. **(H)** Quantification of microglial cell number at increasing distances from 6E10^+^ amyloid plaques shown as a percentage relative to WT in 9-month-old mice. Non-parametric analysis of longitudinal data in factorial experiments followed by a post-hoc Dunn’s test. Significant genotype-by-distance interaction: *F*(5, 50) = 7.242, *P* < 0.0001. WT versus NL-G-F: plaque *P* = 0.011, 0–10 µm *P* = 0.012, 30–40 µm *P* = 0.031, 40–50 µm *P* = 0.02. *n* = 6 per genotype. **(I)** Quantification of microglia coverage at increasing distances from 6E10^+^ Aβ plaques shown as a percentage relative to WT in 9-month-old mice. Repeated measures two-way ANOVA followed by a post-hoc Tukey test. Significant genotype-by-distance interaction: *F*(5, 50) = 110.7, *P* < 0.0001. WT versus NL-G-F: plaque *P* < 0.0001, 0–10 µm *P* = 0.005. *n* = 6 per genotype. For immunostaining 2–3 brain slices were quantified per animal. Images are presented as a z-stack. *P < 0.05, **P < 0.01, **** *P* < 0.0001. Data are represented as mean ± SEM. Each data point represents one animal. DAPI, 4′,6-diamidino-2-phenylindole; IBA1, ionized calcium-binding adaptor molecule 1; NS, not significant; WT, wild-type.

Confocal microscopy images of microglial cells labelled with the microglia marker IBA1, showed that these cells evenly covered the SR of the hippocampus in both WT and NL-G-F 2-month-old mice (Fig. 3A). At this age, microglial cell number, coverage and IBA1 intensity were the same between NL-G-F and WT mice (Fig. 3B and C) (Supplementary Fig. 2A). Using a similar approach to that employed to evaluate synapses, we quantified microglial cell parameters (number, coverage, intensity) in 5- and 9-month-old mice in 10 µm increments from the edge of Aβ plaques, up to 50 µm away using concentric circles (Supplementary Fig. 2G), but this time we also quantified within the plaque. We observed a 4-fold increase in microglial cell number (Fig. 3D and E), a 3-fold increase in coverage (Fig. 3D and F), and a 2.3-fold increase in intensity (Supplementary Fig. 2B) within the plaque. In addition, a significant increase was observed in all parameters at distances proximal to plaques (up to 30 µm away) in 5-month-old NL-G-F mice compared to WT controls (Fig. 3E and F; Supplementary Fig. 2B). At 9 months, the magnitude of the effect on microglial cell number, coverage, and IBA1 intensity was more pronounced than at 5 months (Fig. 3G–I). Here, a 13-fold increase in microglial cell number (Fig. 3G and H), a 4-fold increase in coverage (Fig. 3G and I), and a 3-fold increase in IBA1intensity (Supplementary Fig. 2C) was observed within plaques in NL-G-F mice compared to WT controls. A significant increase in these parameters was also observed up to 10 µm away from plaques, but interestingly we no longer observed differences at 20 µm or 30 µm away from plaques (Fig. 3H and I; Supplementary Fig. 2C). However, intriguingly an increase in microglial number from 30 to 50 µm was observed (Fig. 3H).Although changes in microglial cell number, coverage or IBA1 intensity provide valuable insight, these changes might not fully reflect all the changes that occur in microglial cells in NL-G-F mice. We therefore used RT-qPCRs as a molecular approach to further examine possible changes in microglia. We specifically selected several microglial markers, *Aif1* (encoding for IBA1), *Cd68*, *Cx3cr1*, *Itgam* (encoding for CD11b), *P2ry12*, *Tmem119*, and *Trem2*, that have been shown to be affected in Alzheimer’s disease.^37-39^ At 2 months, we found a small but statistically significant elevation in the mRNA levels for *Cx3cr1* in NL-G-F mice, whereas the remaining microglial markers were not affected (Supplementary Fig. 3A). At 5 months, however, we observed a significant increase in the expression levels of *Cd68* and *Trem2* (Supplementary Fig. 3A). At 9 months, we observed changes in more markers such as *Aif1*, *Cd68*, *Cx3cr1*, *Itgam*, *P2ry12* and *Trem2* (Supplementary Fig. 3A). In addition, the magnitude of the changes between 5 and 9 months was more marked. For example, *Trem2* increased by 1.8 fold at 5 months but almost 5-fold at 9 months. Taken together, our data shows clear and progressive alterations in microglia in NL-G-F mice, with cellular changes occurring particularly around Aβ plaques.

### Astrocyte changes in the NL-G-F mouse model

Analyses of astrocytes have demonstrated that these glial cells exhibit profound alterations in Alzheimer’s disease brains of humans and animal models.^40-42^ However, the changes in these cells during the progression of the condition have not been well characterized. Here we examined astrocytes labelled with GFAP in the CA1 SR hippocampus of NL-G-F mice and WT controls at 2, 5, and 9 months of age, when we observed a gradual progression of excitatory synapse loss in this model. As described for microglia, we quantified astrocyte parameters (coverage, and GFAP intensity) in 10 µm increments from the edge of Aβ plaques, up to 50 µm away using concentric circles, including within the plaque itself. For astrocyte cell number, however, we quantified within the plaque, at 0–25 µm and at 25–50 µm from the plaque using concentric circles (Supplementary Fig. 2H). We took this approach because astrocytes are larger cells than microglia and therefore quantification within 10 µm increments becomes challenging to accurately count cell number.

**Figure 4:**
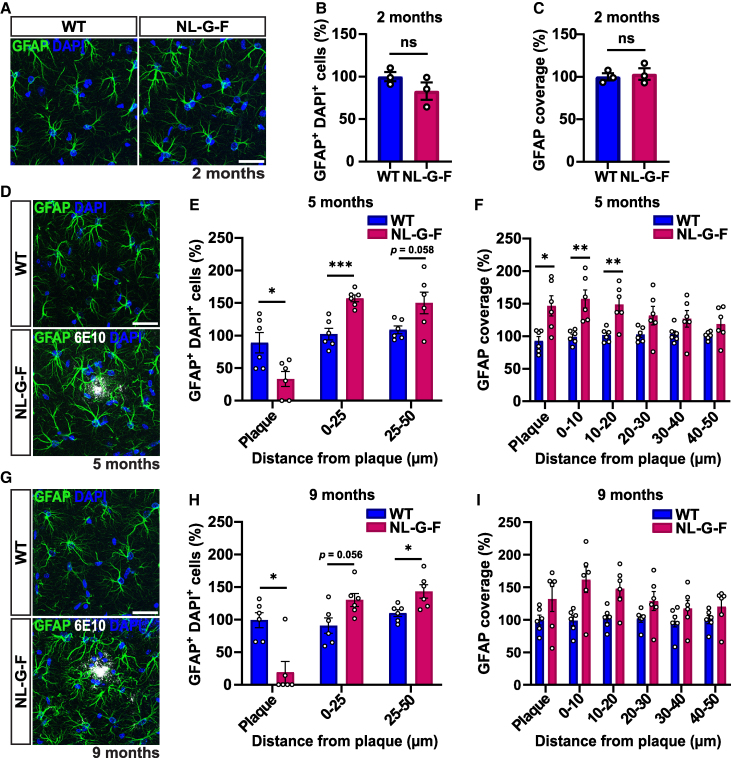
**Changes in astrocyte number and coverage in the hippocampus of NL-G-F mice with age. (A)** Confocal representative images of astrocytes from the CA1 SR of 2-month-old WT and NL-G-F mice. Scale bar 30 µm. (**B-C)** Quantification of astrocyte number (**B**) and coverage (**C**) shown as a percentage relative to WT in 2-month-old mice. Two-tailed unpaired *t*-test. *n* = 3 per genotype. **(D)** Confocal representative images of astrocytes around 6E10^+^ Aβ plaques (white) from the CA1 SR of 5-month-old WT and NL-G-F mice. Scale bar 30 µm. **(E)** Quantification of astrocyte number at increasing distances from 6E10^+^ Aβ plaques shown as a percentage relative to WT in 5-month-old mice. Repeated measures two-way ANOVA followed by a post-hoc Tukey test. Significant genotype-by-distance interaction: *F*(2, 20) = 13.45, *P* = 0.0002. WT versus NL-G-F: plaque *P* = 0.018, 0–25 µm *P* = 0.0007. *n* = 6 per genotype. **(F)** Quantification of astrocyte coverage at increasing distances from 6E10^+^ Aβ plaques shown as a percentage relative to WT in 5-month-old mice. Repeated measures two-way ANOVA followed by a post-hoc Tukey test. Significant genotype-by-distance interaction: *F*(5, 50) = 5.81, *P* = 0.0003. WT versus NL-G-F: plaque *P* = 0.015, 0–10 µm *P* = 0.007, 10–20 µm *P* = 0.0098. *n* = 6 per genotype. **(G)** Confocal representative images of astrocytes around 6E10^+^ Aβ plaques from the CA1 SR of 9-month-old WT and NL-G-F mice. Scale bar 30 µm. **(H)** Quantification of astrocyte number at increasing distances from 6E10^+^ Aβ plaques shown as a percentage relative to WT in 9-month-old mice. Non-parametric analysis of longitudinal data in factorial experiments followed by a post-hoc Dunn’s test. Significant genotype-by-distance interaction: *F*(2, 20) = 14.74, *P* < 0.0001. WT versus NL-G-F: plaque *P* = 0.022, 25–50 µm *P* = 0.037. *n* = 6 per genotype. (**I)** Quantification of astrocyte coverage at increasing distances from 6E10^+^ amyloid plaques shown as a percentage relative to WT in 9-month-old mice. Non-parametric analysis of longitudinal data in factorial experiments followed by a post-hoc Dunn’s test. No significant interaction. *n* = 6 per genotype. For immunostaining 2–3 brain slices were quantified per animal. Images are presented as a z-stack. *P < 0.05, **P < 0.01, ***P < 0.001. Data are represented as means ± SEM. Each data point represents one animal. DAPI, 4′,6-diamidino-2-phenylindole; GFAP, glial fibrillary acidic protein; NS, not significant; WT, wild-type.

At 2 months, we found no differences in the number (DAPI positive GFAP labelled cells in each region of interest), coverage (GFAP positive area within a given region of interest), or mean intensity of GFAP signal, in astrocytes between WT and NL-G-F mice (Fig. 4A–C; Supplementary Fig. 2D). Interestingly, at 5 months, a significant decrease in the number of astrocytes within plaques was observed (Fig. 4D and E). However, a clear increase in astrocyte number in the immediate peri-plaque space (0–25 µm) and a trend towards an increase further away from plaques (25–50 µm) was evident (Fig. 4D and E). In addition, we observed a significant increase in astrocyte coverage within Aβ plaques and up to 20 µm away from plaques (Fig. 4D and F) and a significant increase in GFAP intensity 0–10 µm away from plaques in 5-month-old NL-G-F mice compared to WT controls (Supplementary Fig. 2E). Moreover at 9 months of age, a similar pattern emerged with a decrease in the number of astrocytes within plaques, but a trend to increase at 0–25 µm away from plaques and an increase in number further away at 25–50 µm from plaques in NL-G-F mice compared to WT mice (Fig. 4G and H). Interestingly, we found no differences in astrocyte coverage (Fig. 4G and I) at 9 months, but a significant increase in GFAP intensity at 0–20 µm from the plaque but not within plaques in NL-G-F mice when compared to WT controls (Supplementary Fig. 2F).

We then examined the expression of astrocyte markers using RT-qPCR. We measured the mRNA levels for *Chi3l1* (also known as YKL-40), *Gfap*, *Lcn2*, *Slc1a2* (encoding for GLT-1), *Sparcl1* (encoding for Hevin), *Thbs1* and *Thbs2*, genes that regulate synapse number and/or are affected in Alzheimer’s disease.^42-44^ At 2 months of age, we observed a small increase in the expression of *Chi3l1* and *Slc1a2* but a decreased expression of *Thbs1* and *Thbs2* in the hippocampus of NL-G-F mice when compared to control WT mice (Supplementary Fig. 3B). At 5 months, we found a significant increase in *Gfap* mRNA levels, whilst *Slc1a2*, *Sparcl1,* and *Thbs2* expression were significantly decreased (Supplementary Fig. 3B). At 9 months, the expression of *Gfap* and *Lcn2* was markedly increased when compared to WT controls and to 5 months (Supplementary Fig. 3B). Interestingly, *Chi3l1* expression was increased again at 9 months, whilst *Slc1a2* and *Sparcl1* remained decreased in NL-G-F mice (Supplementary Fig. 3B). These results demonstrate dynamic changes in astrocytic gene expression across different ages with a marked increase in the magnitude of the effect in some markers in NL-G-F mice with age.

### Changes in inflammatory markers in the NL-G-F mouse model

We also investigated inflammatory markers in the hippocampus of NL-G-F mice compared to WT controls using RT-qPCR. At 2 months, complement marker *C3*, pro-inflammatory cytokine *Il-1β* and pro-inflammatory receptor *Tlr4*, were significantly decreased in NL-G-F mice (Supplementary Fig. 3C). At 5 months, the observed decreased expression of *Tlr4* was sustained, with the complement factor *C1qbp*, also showing a decreased expression in NL-G-F mice (Supplementary Fig. 3C). In contrast, *Tnf-α* mRNA expression was increased in NL-G-F mice at 5 months (Supplementary Fig. 3C). At 9 months, *Tnf-α* was significantly increased in NL-G-F mice, accompanied by an increase in *Il-1β* and complement factor, *C1qa* (Supplementary Fig. 3C).

### Characterization of microglia and astrocytes around different types of Aβ plaques

It is well documented that Aβ aggregates form different types of plaques in the brain.^45,46^ For simplification and based on morphological appearance, we broadly categorized plaques, based on 6E10 staining in the SR CA1 hippocampus of NL-G-F mice, into diffuse and non-diffuse plaques (Supplementary Fig. 4A and B). In diffuse plaques, Aβ loosely aggregates, forming plaques with no clear borders. In humans, these plaques are not associated with cognitive decline and might represent early stages of plaque formation.^45^ In contrast, non-diffuse plaques (including dense-core plaques), have a more defined border and typically contain a core comprised of compact Aβ aggregates. These plaques are associated with cognitive decline in humans and might represent more mature plaques.^47,48^ To identify non-diffuse plaques, NL-G-F brain slices were labelled with 6E10 and Thioflavin-S (Thio-S), the latter which labels dense-core plaques (Supplementary Fig. 4C). Comparing the distribution of diffuse and non-diffuse plaques at 5- and 9-months in NL-G-F mice, we found that 60% of plaques were diffuse at 5 months but this percentage decreased to 31% at 9 months (Supplementary Fig. 4D). Thus, an increased proportion of non-diffuse plaques was observed with age in NL-G-F mice. Furthermore, we quantified the average size of plaques in NL-G-F mice at 5- and 9-months, defining size by area (µm^2^). However, we found no differences in plaque size between the two timepoints (Supplementary Fig. 4E).

We then evaluated microglial and astrocyte cell number, coverage, and intensity at different distances around these two types of Aβ plaques at 5- and 9-months of age in the NL-G-F mice. We found no differences between diffuse and non-diffuse plaques for all microglial cell parameters at both ages (Supplementary Fig. 4F–K). However, for astrocytes at 5 months, we found a trend towards an increase in their number close to non-diffuse plaques (0–25 µm) and a significant increase in coverage and GFAP mean intensity from 10 µm away from plaques, except 30–40 µm, in non-diffuse compared to diffuse plaques (Supplementary Fig. 5A–C). In contrast, at 9 months this effect is no longer observed with no significant differences found between diffuse and non-diffuse plaques across all astrocyte parameters (Supplementary Fig. 5D–F). These results suggest that microglial cells and astrocytes do not exhibit clear differential changes between diffuse and non-diffuse plaques in the NL-G-F model.

## Discussion

In Alzheimer’s disease, synapse loss correlates with cognitive decline. Over the past two decades, studies have examined the cellular and molecular mechanisms driving synapse dysfunction. Indeed, it has been demonstrated that Aβ disrupts neuronal signalling crucial for synapse integrity, while glial cell changes can also affect synapses. However, whether glial alterations precede, coincide with, or follow synapse loss remains unclear.

To address this outstanding question, we evaluated NL-G-F mice, an Alzheimer’s disease knock-in mouse model,^27^ at different ages. At 2 months, when very few or no plaques are present, inhibitory synapses transiently increased. By 5 months, excitatory synapses decreased near plaques, which is exacerbated by 9 months. Thus, excitatory synapse loss in the hippocampus of NL-G-F mice is progressive and more evident near Aβ plaques, as we previously reported in 7-month-old NL-G-F mice^31^ and is consistent with other Alzheimer’s disease mouse models and human brains.^8,10^ Moreover, reductions in the optical density of pre- and post-synaptic markers in the hippocampus have been observed in 7-month-old NL-G-F mice.^33^ The early transient rise in inhibitory synapses, also reported in APP/PS1 mice,^49,50^ may reflect a homeostatic response to excitatory synapse dysfunction. Supporting this hypothesis, electrophysiological studies in 2-month-old NL-G-F mice showed an increased release probability at excitatory synapses.^28^ However, further studies are needed to explore this interesting possibility. Differences in the direction and timing of excitatory and inhibitory synapse changes might suggest distinct cellular or molecular mechanisms controlling these processes.

Does disease progression affect glial cells in NL-G-F mice? Microglial cells have been strongly implicated in Alzheimer’s disease pathogenesis and are thought to contribute to synapse vulnerability.^20,22,51^ We therefore analysed microglia across different ages in NL-G-F mice. At the cellular level, we found a marked increase in all microglial parameters (number, coverage, and IBA1 intensity) around plaques in NL-G-F mice only from 5 months, which were exacerbated at 9 months. In line with our work, other studies have documented similar findings in microglial cell number specifically around Aβ plaques in the hippocampus of *NL-F* mice, a related KI Alzheimer’s disease model,^52^ and also in the human Alzheimer’s disease brain.^53,54^ Moreover, other studies have reported an increase in microglial number and coverage in the hippocampus of NL-G-F mice, evident from 9 months onwards.^28,33,55^ Notably, at 5 months, we observed an elevation in microglial cell number up to 20 µm in distance away from plaques. Interestingly, at 9 months, we did not detect a change between 10 and 20 µm from plaques, but a significant increase in microglial cell number inside plaques and immediately proximal to plaques (up to 10 µm). These findings suggest a possible migration of microglia towards Aβ plaques with age.

Interestingly, analyses of microglial markers by RT-qPCR demonstrated early molecular changes in these cells, with an increase in *Cx3cr1* at 2 months in NL-G-F mice.^38^ Notably, at 5 and 9 months, the marker, *Cd68*, which commonly identifies phagocytic microglia,^39^ was markedly increased in NL-G-F mice compared to controls. Moreover, *Trem2*, a key regulator of microglial function and important for microglial response to Aβ plaques,^56^ was also markedly increased in 5- and 9-month-old NL-G-F mice. Similarly, others have reported increased *Trem2* expression in the hippocampus of 9-month-old NL-G-F mice.^28^ These results suggest a progressive increase in active and phagocytic microglia, consistent with results from human Alzheimer’s disease samples.^39,57^

Astrocytes are also implicated in Alzheimer’s disease, although their contribution to the disease pathogenesis is less clear. We therefore analysed astrocyte parameters (number, coverage, and GFAP mean intensity) in NL-G-F mice. Like microglia, we observed clear changes in all astrocyte parameters from 5 months, but they were not exacerbated at 9 months. Notably, astrocyte number was increased around Aβ plaques (<50 µm) in NL-G-F mice at 5 and 9 months. Consistent with our findings, other studies have found a significant increase in astrocyte number and/or coverage at <50 µm from plaques in both human and mouse models of Alzheimer’s disease.^52-54^ Moreover, an increase in astrocyte process area and length and astrocyte coverage has been observed in the hippocampus of 12- and 18-month-old NL-G-F mice compared to WT, respectively.^33,55^ In contrast, some studies in Alzheimer’s disease mouse models, including NL-G-F mice, reported that astrocyte number in the hippocampus does not change compared to WT.^33,58^ Intriguingly, at 5 and 9 months, we found that astrocyte number (GFAP and DAPI positive cells) was decreased inside plaques, whilst coverage, in 5-month NL-G-F mice, was significantly increased inside plaques. These results suggest an increase in astrocytic processes extending into the plaque. Consistent with this result, it has been suggested that astrocyte cell bodies remain largely immobile around plaques, but their processes orient towards plaques.^59^

RT-qPCR analyses showed early (2 months) molecular changes in astrocyte markers, notably an increased expression in *Slc1a2*, which encodes for GLT-1, a glutamate transporter expressed in astrocytes that regulates glutamate levels.^60^ Interestingly, this earlier increase in *Slc1a2* expression was not sustained, as its expression was significantly decreased in NL-G-F mice from 5 months. Consistent with this result, decreased expression of GLT-1 has been observed in Alzheimer’s disease.^61^ Moreover, loss-of-function of GLT-1 in an Alzheimer’s disease mouse model exhibited accelerated cognitive deficits.^62^ Thus, the increase in *Slc1a2* expression suggests a possible compensatory mechanism in response to synaptic insult and efforts to preserve cognitive function. Other interesting astrocyte markers are THBS1/2, that encode the astrocyte-secreted proteins, Thrombospondin 1 and 2, that promote excitatory synapse formation in the central nervous system.^63,64^ In NL-G-F mice at 2 months, the expression of *Thbs1* and *Thbs2* was decreased. Indeed, THBS1 is decreased in the human Alzheimer’s disease brain, its gain-of-function prevents Aβ-mediated synaptic toxicity.^65^ Therefore, the early downregulation of *Thbs1/2* in NL-G-F mice might indicate the start of excitatory synapse vulnerability in these mice. At 5 months, the decreased expression of *Thbs2* was maintained with a concomitant decrease in the expression of *Sparcl1*, a gene also implicated in synapse formation.^66^ Given the role of THBSs and SPARCL1 on synapses, their decrease could contribute to synapse loss in these mice at 5 months. Importantly, at this age, a significant increase in *Gfap* mRNA levels was detected, coinciding with the histological changes in astrocytes in NL-G-F mice. At 9 months, the effect on these markers was exacerbated except for *Thbs2* and the addition of a marked increase in *Lcn2*, a gene that encodes a protein implicated in neuroinflammation.^43^

Several inflammatory markers were also examined at the molecular level. Interestingly, we found a decrease in the expression of several genes at 2 months in NL-G-F mice including *C3, Il-1β,* and *Tlr4.* However, by 9 months, a clear increase in several inflammatory markers was evident including, *C1qa,* and *Tnf-α,* in NL-G-F mice compared to controls. C1qa forms part of the C1q complex that initiates the classical complement pathway and mediates synapse engulfment in Alzheimer’s disease.^22^ Furthermore, TNF-α, a pro-inflammatory cytokine that is elevated in Alzheimer’s disease, can trigger the production of other inflammatory molecules and is associated with accelerated disease progression.^67^ Together, our results suggest an increase in phagocytic microglia and inflammatory response with age in NL-G-F mice.

A strength of our study is that we present a comprehensive timeline of key events unfolding in a powerful Alzheimer’s disease mouse model that expresses mutant human APP at endogenous levels. However, the NL-G-F model lacks tau pathology. Therefore, the contribution of tau or other Alzheimer’s disease pathologies such as TDP-43, were not examined given the model employed. Tau is particularly relevant because it can contribute to synaptic dysfunction, gliosis and inflammation in Alzheimer’s disease.^68,69^ Another limitation of our study is the lack of analysis of sex as a biological variable, which has been reported in the NL-G-F model.^55,70-72^

**Figure 5:**
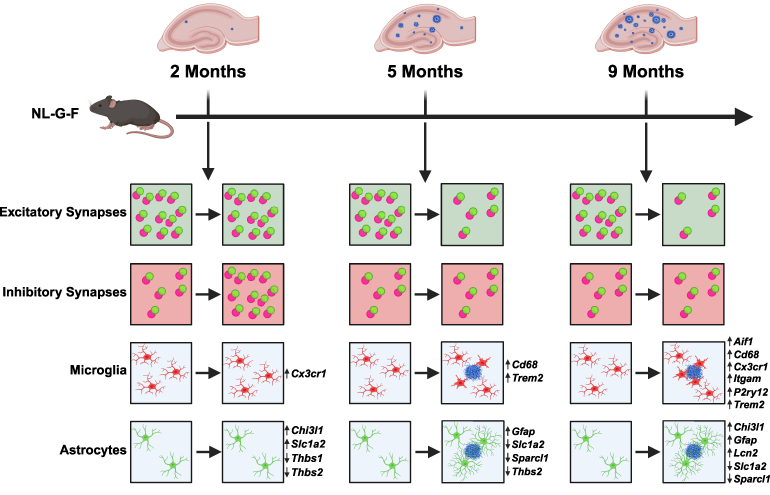
**Schematic overview of the findings.** A schematic overview of our findings in NL-G-F mice at 2, 5, and 9 months of age compared to WT controls. At 2 months, we observed an increase in the number of inhibitory synapses, but no changes in the number of excitatory synapses or in microglial cell or astrocyte number, coverage, or IBA1 or GFAP intensity, respectively. By 5 months, no change in inhibitory synapses were observed, but a decrease in excitatory synapse number, with concomitant changes in the number, coverage, and intensity of both microglia and astrocytes were observed near Aβ plaques. At 9 months, inhibitory synapse number remained unchanged, whereas the loss of excitatory synapses and changes in microglia around Aβ plaques was more pronounced than at 5 months. Slight changes were observed in astrocytes from 5 months onward. Molecular changes were observed in both microglia and astrocyte markers across all age groups. Created in BioRender. Salinas, P. (2025) https://BioRender.com/hy2q4nl. *Cx3cr1*, CX3C motif chemokine receptor 1; *Cd68*, cluster of differentiation 68; *Trem2*, triggering receptor expressed on myeloid cells 2; *Aif1*, allograft inflammatory factor 1; *Itgam*, integrin alpha M; *P2ry12*, purinergic receptor P2Y G-protein coupled 12; *Chi3l1*, chitinase-3-like protein 1; *Slc1a2*, solute carrier family 1 member 2; *Thbs1*, thrombospondin 1; *Thbs2*, thrombospondin 2; *Gfap*, glial fibrillary acidic protein; *Sparcl1*, SPARC-like protein 1; *Lcn2*, lipocalin 2.

In summary, our study of NL-G-F mice across different ages defines a temporal sequence linking changes in synapse number, glial cells, and Aβ pathology (Fig. 5). We demonstrate a clear and progressive loss of excitatory synapses following plaque deposition in the hippocampus, with no histological changes in microglia or astrocytes preceding excitatory synapse loss. At early stages, however, inhibitory synapses were transiently increased before significant deposition of Aβ was observed. This increase was accompanied by molecular changes in astrocytes and microglia. Thus, inhibitory synapse number and glial molecular alterations represent the earliest detectable changes in NL-G-F mice, preceding excitatory synapse loss, overt cellular changes in glial populations, and substantial plaque deposition.

## Supplementary Material

fcaf484_Supplementary_Data

## Data Availability

All data reported is available in the main text and/or the Supplementary Materials. Any additional information is available upon request.
