# Supplementary material for: Progressive changes in synapses and glial cells in AppNL-G-F mice, a model of Alzheimer’s disease
Source: Brain Commun. 2025 Dec 12;8(1):fcaf484. doi: 10.1093/braincomms/fcaf484 (PMC12957919; doi:10.1093/braincomms/fcaf484)
Supplement: fcaf484_Supplementary_Data [file fcaf484_Supplementary_Data.pdf]

Supplementary Figure 1

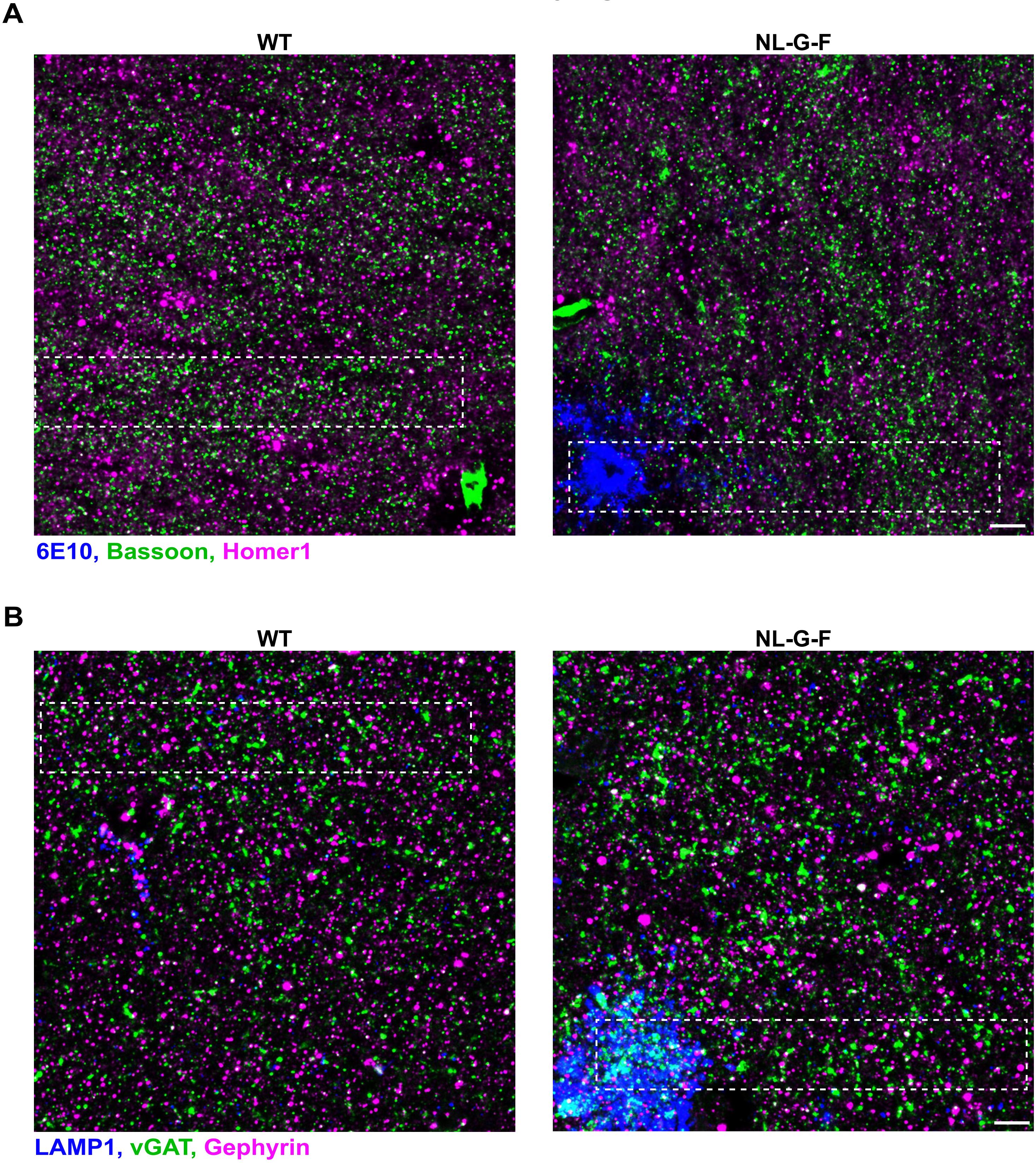

Supplementary Figure 1: Excitatory and inhibitory synapses around Aβ plaques

A) Low magnification z-stack confocal images of excitatory synapses (bassoon in green and homer1 in magenta) around a 6E10-labelled Aβ plaque (blue) from the CA1 SR of 5-month-old WT and NL-G-F mice. The white dotted box marks the region used for synapse quantification. Scale bar 5μm. **B)** Low magnification z-stack confocal images of inhibitory synapses (vGAT in green and gephyrin in magenta) around a LAMP1-labelled Aβ plaque (blue) from the CA1 SR of 5-month-old WT and NL-G-F mice. The white dotted box marks the region used for synapse quantification. Scale bar 5μm. Homer1, homer scaffold protein 1; LAMP1, lysosomal-associated membrane protein 1; vGAT, vesicular GABA transporter; WT, wild-type.

Supplementary Figure 2

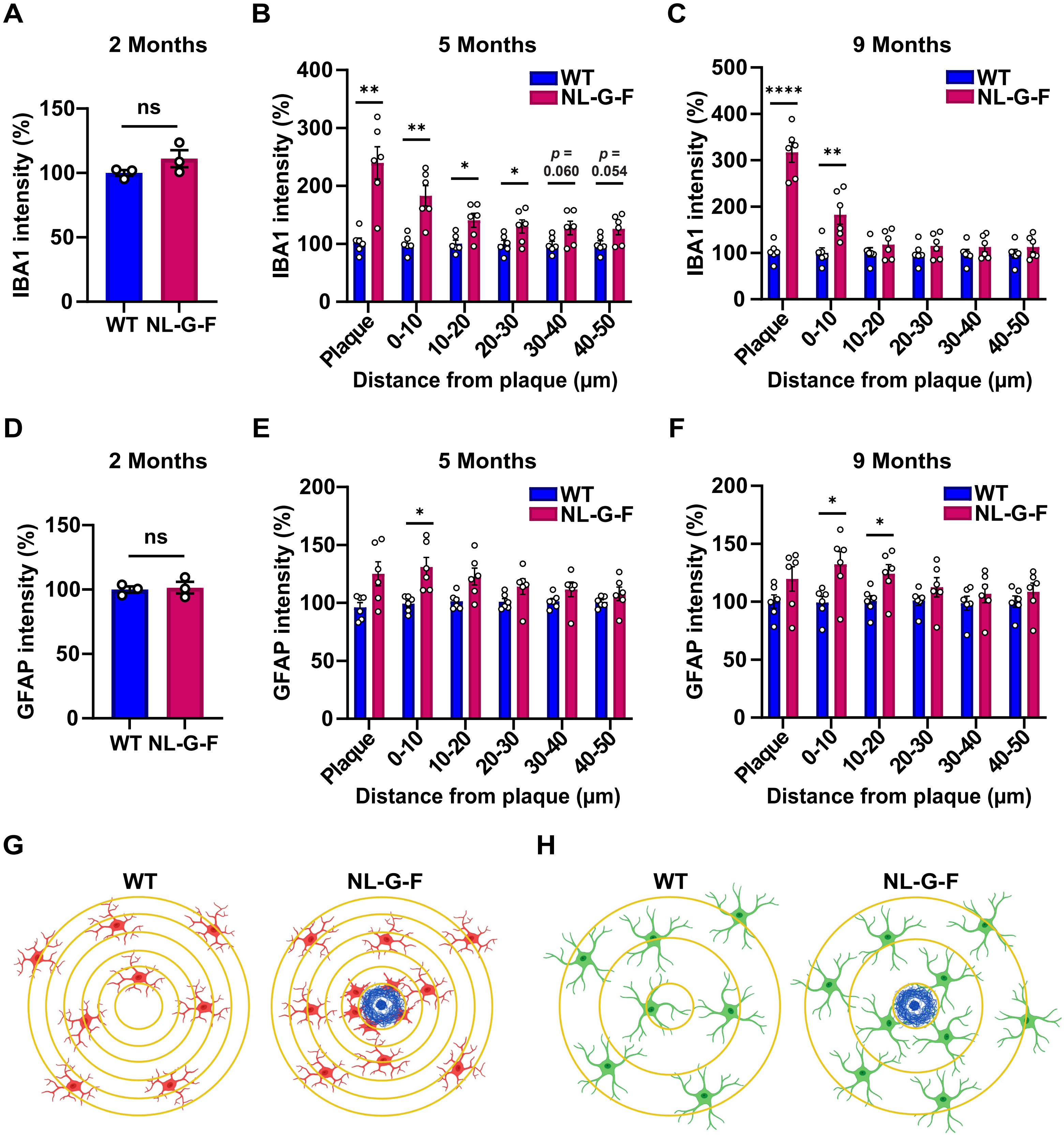

Supplementary Figure 2: Progressive cellular changes in microglia and astrocytes with age

A) Quantification of IBA1 mean intensity shown as a percentage relative to WT in 2-month-old WT and NL-G-F mice. Two-tailed unpaired t-test. n=3 per genotype. **B)** Quantification of IBA1 mean intensity at increasing distances from 6E10+ Aβ plaques shown as a percentage relative to WT in 5-month-old mice. Repeated measures two-way ANOVA followed by a post-hoc Tukey test. Significant genotype-by-distance interaction: F (5, 50) = 26.87, p<0.0001. WT vs NL-G-F: Within Aβ plaques p=0.003, 0-10μm p=0.0039, 10-20μm p=0.018, 20-30μm p=0.049. n=6 per genotype. **C)** Quantification of IBA1 mean intensity at increasing distances from 6E10+ Aβ plaques shown as a percentage relative to WT in 9-month-old mice. Repeated measures two-way ANOVA followed by a post-hoc Tukey test. Significant genotype-by-distance interaction: F (5, 50) = 107.1, p<0.0001. WT vs NL-G-F: Within Aβ plaques p<0.0001, 0-10μm p=0.008. n=6 per genotype. **D)** Quantification of GFAP mean intensity shown as a percentage relative to WT in 2-month-old WT and NL-G-F mice. Two-tailed unpaired t-test. n=3 per genotype. **E)** Quantification of GFAP mean intensity at increasing distances from 6E10+ Aβ plaques shown as a percentage relative to WT in 5-month-old mice. Non-parametric analysis of longitudinal data in factorial experiments followed by a post-hoc Dunn's test. No significant interaction. WT vs NL-G-F: 0-10μm p=0.012. n=6 per genotype. **F)** Quantification of GFAP mean intensity at increasing distances from 6E10+ Aβ plaques shown as a percentage relative to WT in 9-month-old mice. Repeated measures two-way ANOVA followed by a post-hoc Tukey test. Significant genotype-by-distance interaction: F (5, 50) = 6.70, p<0.0001. WT vs NL-G-F: 0-10μm p=0.029, 10-20μm p=0.033. n=6 per genotype. **G-H)** Schematic diagrams depicting how microglial **(G)** and astrocyte **(H)** cell number was analysed using concentric circles in WT and NL-G-F mice. Microglial and astrocyte coverage and intensity of their respective markers, IBA1 and GFAP, were also analysed according to panel G. Created in BioRender. Salinas, P. (2025) https://BioRender.com/7uz1h1q (G) and https://BioRender.com/dbqoih4 (H). For immunostaining 2-3 brain slices were quantified per animal. *p<0.05, **p<0.01, ****p<0.0001. Data are represented as mean ± SEM. Each data point represents one animal. IBA1, ionised calcium-binding adaptor molecule 1; GFAP, glial fibrillary acidic protein; NS, not significant; WT, wild-type.

Supplementary Figure 3

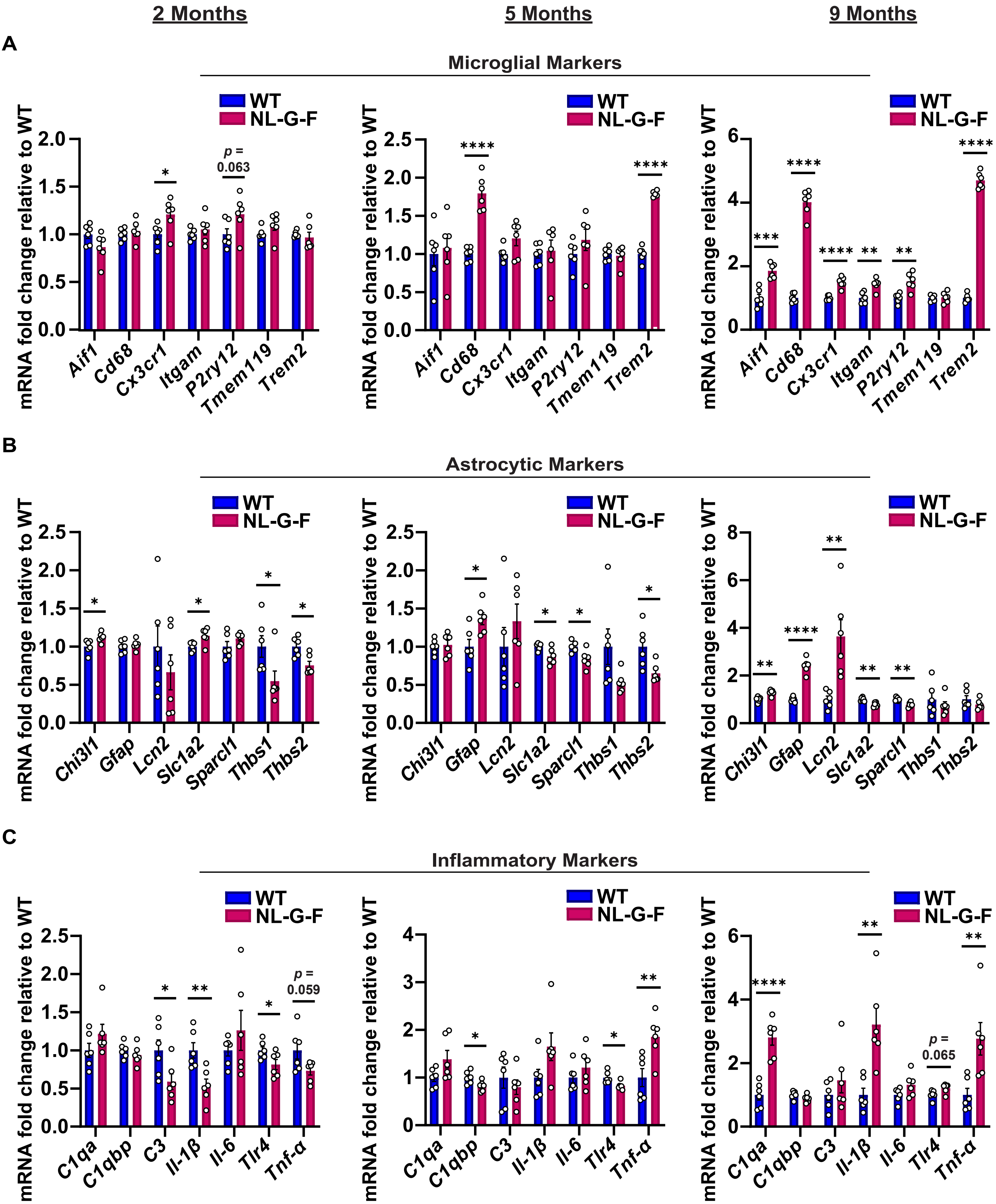

Supplementary Figure 3: Changes in microglial, astrocytic, and inflammatory gene expression in the hippocampus of NL-G-F mice with age

A) qPCRs showing mRNA fold changes relative to WT for microglia related genes in the hippocampus of 2-, 5- and 9-month-old WT and NL-G-F mice, respectively. Two-tailed unpaired t-test. 2 months: *Cx3cr1* p=0.032; *P2ry12* p=0.063. 5 months: *Cd68* and *Trem2* p<0.0001. 9 months: *Aif1* p=0.0002; *Cd68* p<0.0001; *Cx3cr1* p<0.0001; *Itgam* p=0.002; *P2ry12* p=0.002; *Trem2* p<0.0001. n=6 per genotype. **B)** qPCRs showing mRNA fold changes relative to WT for astrocyte related genes in the hippocampus of 2-, 5- and 9-month-old WT and NL-G-F mice, respectively. Two-tailed unpaired Mann-Whitney U test was performed for *Thbs1* and *Thbs2* at 2 months. All other markers across all age groups, a two-tailed unpaired t-test was performed. 2 months: *Chi3l1* p=0.019; *Slc1a2* p=0.018; *Thbs1* p=0.026; *Thbs2* p=0.015. 5 months: *Gfap* p=0.014; *Slc1a2* p=0.02; *Sparcl1* p=0.013; *Thbs2* p=0.016. 9 months: *Chi3l1* p=0.001; *Gfap* p<0.0001; *Lcn2* p=0.005; *Slc1a2* p=0.001; *Sparcl1* p=0.002. n=6 per genotype. **C)** qPCRs showing mRNA fold changes relative to WT for inflammatory marker genes in the hippocampus of 2-, 5- and 9-month-old WT and NL-G-F mice, respectively. Two-tailed unpaired Mann-Whitney U test was performed for *C1qa* at 2 months and *Tlr4* at 9 months. All other markers across all age groups, a two-tailed unpaired t-test was performed. 2 months: *C3* p=0.037; *Il-1* β p=0.006;*Tlr4* p=0.047; *Tnf-α* p=0.059. 5 months: *C1qbp* p=0.027; *Tlr4* p=0.016; *Tnf-α* p=0.0096. 9 months: *C1qa* p<0.0001; *Il-1*β p=0.003; *Tlr4* p=0.065; *Tnf-α* p=0.0097. n=6 per genotype. *p<0.05, **p<0.01, ***p<0.001, ****p<0.0001. Data are represented as mean ± SEM. Each data point represents one animal. *Aif1*, allograft inflammatory factor 1; *Cd68*, cluster of differentiation 68; *Cx3cr1*, CX3C motif chemokine receptor 1; *Itgam*, integrin alpha M; *P2ry12*, purinergic receptor P2Y G-protein coupled 12; *Tmem119*, transmembrane protein 119; *Trem2*, triggering receptor expressed on myeloid cells 2; *Chi3l1*, chitinase-3-like protein 1; *Gfap*, glial fibrillary acidic protein; *Lcn2*, lipocalin 2; *Slc1a2*, solute carrier family 1 member 2; *Sparcl1*, SPARC-like protein 1; *Thbs1*, thrombospondin 1; *Thbs2*, thrombospondin 2; *C1qa*, complement C1q A chain; *C1qbp*, complement C1q binding protein; *C3*, complement component 3; *Il-1β*, interlukin-1 beta; *Il-6*, interlukin-6; *Tlr4*, toll-like receptor 4; *Tnf-α,* tumor necrosis factor alpha; WT, wild-type.

Supplementary Figure 4

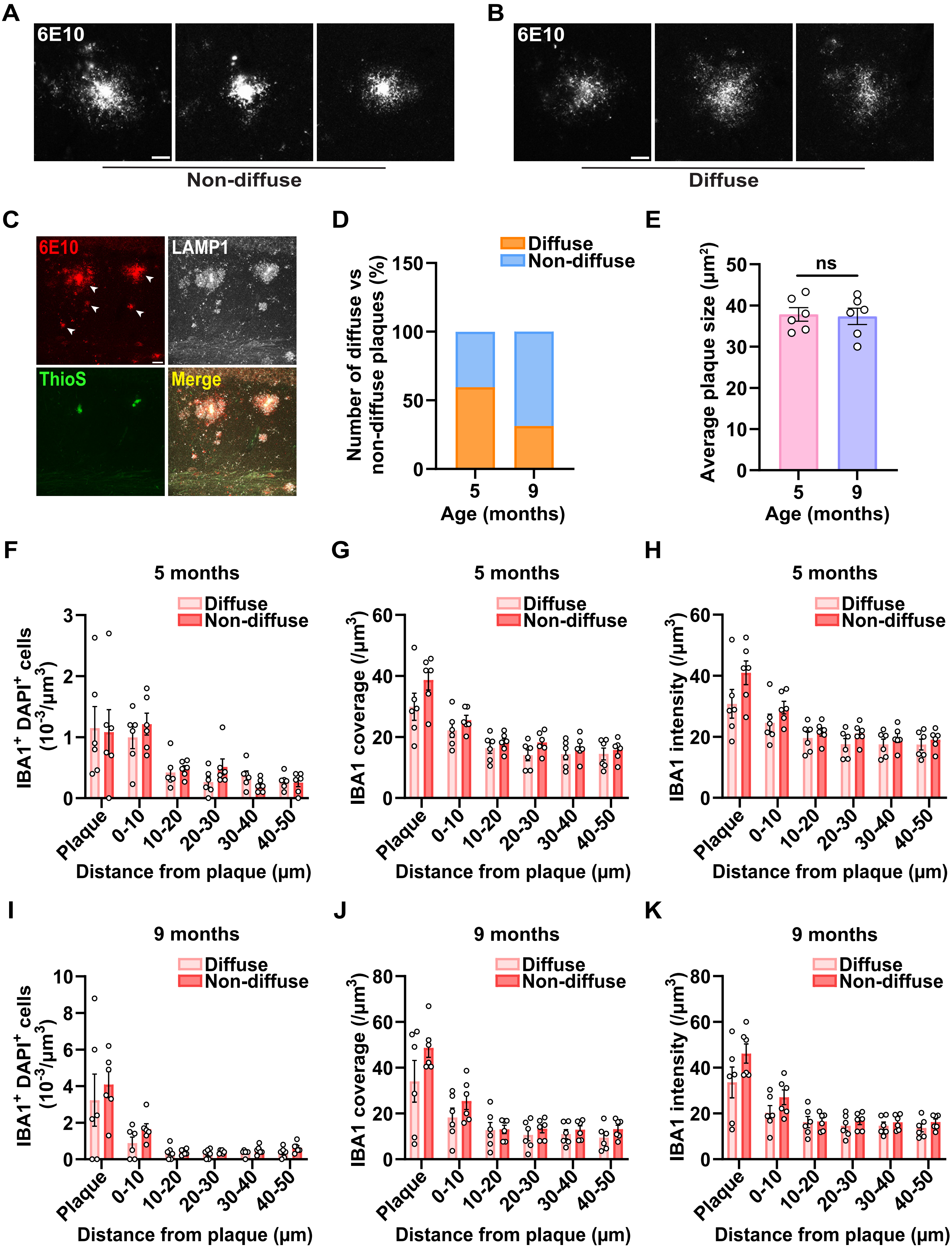

Supplementary Figure 4: Age-related changes in microglia according to Aβ plaque type

A) Confocal representative images of non-diffuse plaques stained with 6E10 in the CA1 SR. Scale bar 10μm. **B)** Confocal representative images of diffuse plaques stained with 6E10 in the CA1 SR. Scale bar 10μm. **C)** Confocal representative images in the CA1 SR, labelled with 6E10+ Aβ plaques (red) (top left), LAMP1 (grey) (top right), Thioflavin-S (green) (bottom left) and a merge image of all three channels (bottom right). Scale bar 20μm. **D)** Quantification of the ratio of diffuse plaques vs non-diffuse plaques normalised to total number of plaques in 5-month-old and 9-month-old NL-G-F mice. n=6 mice per age group. **E)** Quantification of the average size of 6E10+ Aβ plaques, as measured by area in μm2, in 5-month-old and 9-month-old NL-G-F mice. Two-tailed unpaired t-test. n=6 mice per age group. **F)** Quantification of microglia number in diffuse vs non-diffuse plaques at increasing distances from 6E10+ Aβ plaques in 5-month-old NL-G-F mice. Data are presented as average per brain. Non-parametric analysis of longitudinal data in factorial experiments followed by a post-hoc Dunn's test. No significant interaction. n=6 per group. **G)** Quantification of microglia coverage in diffuse vs non-diffuse plaques at increasing distances from 6E10+ Aβ plaques in 5-month-old NL-G-F mice. Data is presented as average per brain. Repeated measures two-way ANOVA followed by a post-hoc Tukey test. No significant interaction. n=6 per group. **H)** Quantification of IBA1 mean intensity in diffuse vs non-diffuse plaques at increasing distances from 6E10+ Aβ plaques in 5-month-old NL-G-F mice. Data are presented as average per brain. Repeated measures two-way ANOVA followed by a post-hoc Tukey test. Significant genotype-by-distance interaction: F (5, 50) = 2.41, p=0.049. n=6 per group. **I)** Quantification of microglia number in diffuse vs non-diffuse plaques at increasing distances from 6E10+ amyloid plaques in 9-month-old NL-G-F mice. Data are presented as average per brain. Non-parametric analysis of longitudinal data in factorial experiments followed by a post-hoc Dunn's test. No significant interaction. n=6 per group. **J)** Microglia coverage in diffuse vs non-diffuse plaques at increasing distances from 6E10+ amyloid plaques in 9-month-old NL-G-F mice. Data are presented as average per brain. Non-parametric analysis of longitudinal data in factorial experiments followed by a post-hoc Dunn's test. No significant interaction. n=6 per group. **K)** IBA1 mean intensity in diffuse vs non-diffuse plaques at increasing distances from 6E10+ Aβ plaques in 9-month-old NL-G-F mice. Data are presented as average per brain. Repeated measures two-way ANOVA followed by a post-hoc Tukey test. No significant interaction. n=6 per group. For immunostaining 2-3 brain slices were quantified per animal. Data are represented as mean ± SEM. Panels E-K, each data point represents one animal. DAPI, 4′,6-diamidino-2-phenylindole; LAMP1, lysosomal-associated membrane protein 1; ThioS, Thioflavin-S; IBA1, ionised calcium-binding adaptor molecule 1; NS, not significant.

Supplementary Figure 5

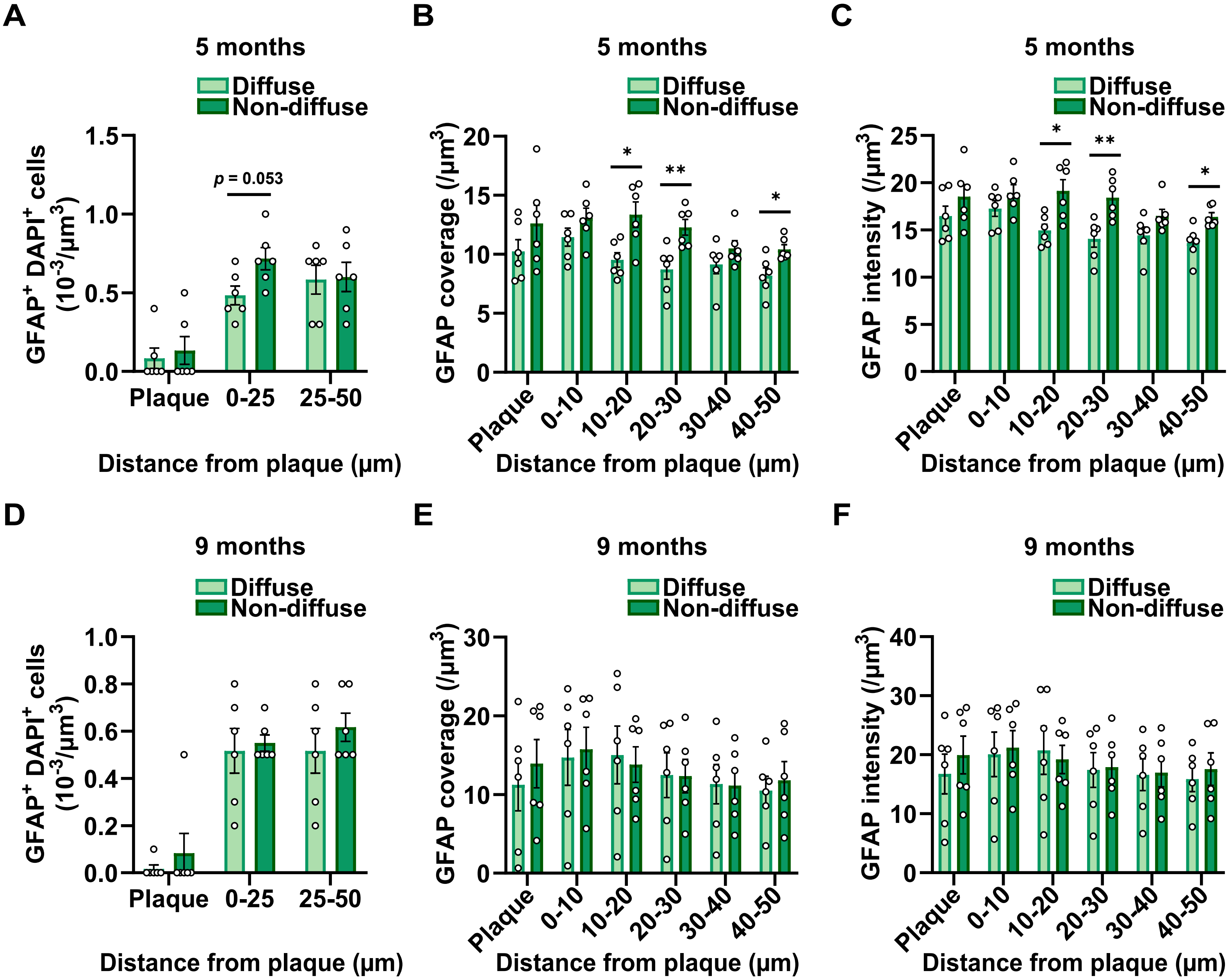

Supplementary Figure 5: Age-related changes in astrocytes according to Aβ plaque type

A) Quantification of astrocyte number in diffuse vs non-diffuse plaques at increasing distances from 6E10+ amyloid plaques in 5-month NL-G-F mice. Data are presented as average per brain. Non-parametric analysis of longitudinal data in factorial experiments followed by a post-hoc Dunn's test. No significant interaction. n=6 per group. **B)** Astrocyte coverage in diffuse vs non-diffuse plaques at increasing distances from 6E10+ amyloid plaques in 5-month NL-G-F mice. Data are presented as average per brain. Repeated measures two-way ANOVA followed by a post-hoc Tukey test. No significant interaction. Diffuse vs non-diffuse: plaque 10-20µm p=0.015, 20-30µm p=0.0078, 40-50µm p=0.018. n=6 per group. **C)** GFAP mean intensity in diffuse vs non-diffuse plaques at increasing distances from 6E10+ Aβ plaques in 5-month NL-G-F mice. Data are presented as average per brain. Repeated measures two-way ANOVA followed by a post-hoc Tukey test. No significant interaction. Diffuse vs non-diffuse: plaque 10-20µm p=0.015, 20-30µm p=0.006, 40-50µm p=0.011. n=6 per group. **D)** Quantification of astrocyte number in diffuse vs non-diffuse plaques at increasing distances from 6E10+ Aβ plaques in 9-month NL-G-F mice. Data are presented as average per brain. Non-parametric analysis of longitudinal data in factorial experiments followed by a post-hoc Dunn's test. No significant interaction. n=6 per group. **E)** Astrocyte coverage in diffuse vs non-diffuse plaques at increasing distances from 6E10+ Aβ plaques in 9-month NL-G-F mice. Repeated measures two-way ANOVA followed by a post-hoc Tukey test. No significant interaction. n=6 per group. **F)** GFAP mean intensity in diffuse vs non-diffuse plaques at increasing distances from 6E10+ Aβ plaques in 9-month NL-G-F mice. Repeated measures two-way ANOVA followed by a post-hoc Tukey test. No significant interaction. n=6 per group. For immunostaining 2-3 brain slices were quantified per animal. *p<0.05, **p<0.01. Data are represented as mean ± SEM. Each data point represents one animal. DAPI, 4′,6-diamidino-2-phenylindole; GFAP, glial fibrillary acidic protein.

Supplementary Material 1

FIJI Macro - Concentric circles around Aβ plaques:

```
function findRoisWithName(roiName) {
	nR = roiManager("Count");
	roiIdx = newArray(nR);
	k=0;
	clippedIdx = newArray(0);

	for (i=0; i<nR; i++) {
		roiManager("Select", i);
		rName = Roi.getName();
		if (matches(rName, roiName) ) {
			roiIdx[k] = i;
			k++;
		}
	}
	if (k>0) {
		clippedIdx = Array.trim(roiIdx,k);
	}

	return clippedIdx;
}

function findRoiWithName(roiName) {
	nR = roiManager("Count");

	for (i=0; i<nR; i++) {
		roiManager("Select", i);
		rName = Roi.getName();
		if (matches(rName, roiName)) {
			return i;
		}
	}
	return -1;
}



setMinAndMax(0, 75);
run("8-bit");

run("Select None");

setAutoThreshold("Default");

setThreshold(45, 255);

setOption("BlackBackground", false);

run("Analyze Particles...", "size=250-Infinity circularity=0.00-1.00 display exclude clear add");

roiManager("Deselect");

roiManager("Select", 0);

run("From ROI Manager");
```

```
lastroi = roiManager("Count")-1;
roiManager("Select", lastroi);
roiManager("Rename", "Plaques");

plaques = findRoiWithName("Plaques");
roiManager("Select", plaques);
run("Enlarge...", "enlarge=10");
roiManager("Add");
lastroi = roiManager("Count")-1;
roiManager("Select", lastroi);
roiManager("Rename", "10um enlargement");

plaques = findRoiWithName("Plaques");
roiManager("Select", plaques);
run("Enlarge...", "enlarge=20");
roiManager("Add");
lastroi = roiManager("Count")-1;
roiManager("Select", lastroi);
roiManager("Rename", "20um enlargement");

plaques = findRoiWithName("Plaques");
roiManager("Select", plaques);
run("Enlarge...", "enlarge=30");
roiManager("Add");
lastroi = roiManager("Count")-1;
roiManager("Select", lastroi);
roiManager("Rename", "30um enlargement");

plaques = findRoiWithName("Plaques");
roiManager("Select", plaques);
run("Enlarge...", "enlarge=40");
roiManager("Add");
lastroi = roiManager("Count")-1;
roiManager("Select", lastroi);
roiManager("Rename", "40um enlargement");

plaques = findRoiWithName("Plaques");
roiManager("Select", plaques);
run("Enlarge...", "enlarge=50");
roiManager("Add");
lastroi = roiManager("Count")-1;
roiManager("Select", lastroi);
roiManager("Rename", "50um enlargement");

um10 = findRoiWithName("10um enlargement");
roiManager("Select", newArray(plaques, um10));
roiManager("XOR");
roiManager("Add");
lastroi = roiManager("Count")-1;
roiManager("Select", lastroi);
roiManager("Rename", "10um Ring");

um20 = findRoiWithName("20um enlargement");
roiManager("Select", newArray(um10, um20));
roiManager("XOR");
roiManager("Add");
lastroi = roiManager("Count")-1;
roiManager("Select", lastroi);
roiManager("Rename", "20um Ring");
```

```
um30 = findRoiWithName("30um enlargement");
roiManager("Select", newArray(um20, um30));
roiManager("XOR");
roiManager("Add");
lastroi = roiManager("Count")-1;
roiManager("Select", lastroi);
roiManager("Rename", "30um Ring");

um40 = findRoiWithName("40um enlargement");
roiManager("Select", newArray(um30, um40));
roiManager("XOR");
roiManager("Add");
lastroi = roiManager("Count")-1;
roiManager("Select", lastroi);
roiManager("Rename", "40um Ring");

um50 = findRoiWithName("50um enlargement");
roiManager("Select", newArray(um40, um50));
roiManager("XOR");
roiManager("Add");
lastroi = roiManager("Count")-1;
roiManager("Select", lastroi);
roiManager("Rename", "50um Ring");

roiManager("Select", newArray(um10, um20, um30, um40, um50));
roiManager("Delete");
;
```

Supplementary Material 2

R Script - Non-parametric analysis of longitudinal data in factorial experiments:

```
library(car)
library(nparLD)
library(tidyr)
library(readxl)

insert here excel file name<- read_excel("insert here file path")
View(file name)

file name$`Mouse ID` <- factor(file name$`Mouse ID`)
insert here excel file name <- gather(file name, distance, synapse, D20:D50, factor_key=TRUE)

var<-file name[,"synapse"]
time<-file name[,"distance"]
group<-file name[,"Genotype"]
subject<-file name[,"Mouse ID"]

f1.ld.f1(y=file name$synapse,time = file name$distance,group=file name$Genotype,subject = file
name$`Mouse ID`)

library(purrr)
library(dplyr)
wilcox_results <- file name %>%
  group_by(Genotype) %>%
  summarise (wilcox_test = list(pairwise.wilcox.test(synapse, distance, p.adjust.method = "bonferroni")))
wilcox_results

wilcox_results %>%
  mutate(p_values = map(wilcox_test, ~ .x$p.value))
wilcox_results$wilcox_test[[1]]$p.value
wilcox_results$wilcox_test[[2]]$p.value

library(ggplot2)

ggplot(file name, aes(x=distance, y=synapse, color=Genotype)) +
  geom_point() +
  geom_line(aes(group=`Mouse ID`), alpha=0.5) +
  theme_minimal() +
  labs(title="Synapse count by Distance from plaques and Genotype", x="Distance from plaques (um)",
y="Synapse count")

file name <- read_excel("file path")
View(file name)
library(dunn.test)
D20 <- dunn.test (file name $D20, file name $Genotype, label= TRUE)
D30 <- dunn.test (file name $D30, file name $Genotype, label= TRUE)
D40 <- dunn.test (file name $D40, file name $Genotype, label= TRUE)
D50 <- dunn.test (file name $D50, file name $Genotype, label= TRUE)
p_values <- c(D20$P, D30$P, D40$P, D50$P)
p_adjusted <- p.adjust(p_values, method = "bonferroni")
print(p_adjusted)
```
